# Assessing COVID-19 Vaccine Effectiveness and Risk Factors for Severe Outcomes through Machine Learning Techniques: A Real-World Data Study in Andalusia, Spain

**DOI:** 10.1007/s44197-024-00298-2

**Published:** 2024-11-11

**Authors:** Álvaro Serrano-Ortiz, Juan Luis Romero-Cabrera, Jaime Monserrat Villatoro, Jaime Cordero-Ramos, Rafael Ruiz-Montero, Álvaro Ritoré, Joaquín Dopazo, Jorge del Diego Salas, Valle García Sánchez, Inmaculada Salcedo-Leal, Miguel Ángel Armengol de la Hoz, Isaac Túnez, Miguel Ángel Guzmán

**Affiliations:** 1 Preventive Medicine and Public Health Unit, Reina Sofía University Hospital, Córdoba, Spain; 2 Preventive Medicine and Public Health Research Group, Maimonides Biomedical Research Institute of Córdoba (IMIBIC), Córdoba, Spain; 3 Preventive Medicine and Public Health Unit, Healthcare Management Area: South of Córdoba, Cabra, Córdoba, Spain; 4 Lipids and Atherosclerosis Unit, Maimonides Biomedical Research Institute of Córdoba (IMIBIC), Reina Sofia University Hospital, University of Córdoba, Córdoba, Spain; 5 CIBEROBN (CIBER in Physiopathology of Obesity and Nutrition), Instituto de Salud Carlos III, Madrid, Spain; 6 Health District of Córdoba and Guadalquivir, Córdoba, Spain; 7 Maimonides Biomedical Research Institute of Córdoba (IMIBIC), Córdoba, Spain; 8 Pharmaceutical Management Department, Extremadura Health Service, Mérida, Spain; 9 Hospital Pharmacy, Virgen Macarena University Hospital, Seville, Spain; 10 Institute of Biomedicine of Seville (IBiS)/University Hospital Virgen del Rocío/CSIC/University of Sevilla, Seville, Spain; 11 Department of Medical and Surgical Sciences, University of Córdoba, Córdoba, Spain; 12 Big Data Department, PMC-FPS, Regional Ministry of Health and Consumer Affairs, Seville, Spain; 13 Computational Medicine Platform, Andalusian Public Foundation Progress and Health-FPS, Seville, Spain; 14 Directorate General of Public Health and Pharmaceutical Regulation, Ministry of Health and Consumer Affairs of the Regional Government of Andalusia, Seville, Spain; 15 Management Directorate of Andalusian Health Service, Ministry of Health and Consumer Affairs of the Regional Government of Andalusia, Seville, Spain; 16 Reina Sofía University Hospital, Córdoba, Spain; 17 Department of Biochemistry and Molecular Biology, University of Córdoba, Córdoba, Spain; 18 General Secretariat of Public Health and Research, Development and Innovation in Health, Ministry of Health and Consumer Affairs of the Regional Government of Andalusia, Seville, Spain; 19 Andalusian Public Healthcare System, Andalusia, Spain

**Keywords:** COVID-19, Epidemiology, Health policy, Cohort study, Vaccines

## Abstract

**Background:**

COVID-19 vaccination has become a pivotal global strategy in managing the pandemic. Despite COVID-19 no longer being classified as a Public Health Emergency of International Concern, the virus continues affecting people worldwide. This study aimed to evaluate risk factors and vaccine effectiveness on COVID-19-related hospital admissions, intensive care unit (ICU) admission and mortality within the Andalusian population throughout the pandemic.

**Methods:**

From March 2020 to April 2022, 671,229 individuals, out of 9,283,485 with electronic health records in Andalusia, experienced SARS-CoV-2 infection and were included in the analysis. Data on demographics, medical history, vaccine administration, and hospitalization records were collected. Associations between medical history, COVID-19 vaccines, and COVID-19 outcomes were assessed.

**Results:**

Our study identified 48,196 hospital admissions, 5,057 ICU admissions, and 11,289 deaths linked to COVID-19. Age, male sex, and chronic diseases were identified as risk factors, while the COVID-19 vaccine demonstrated protective effects, although with reduced effectiveness during the omicron variant period. However, the risk for these outcomes increased over time after receiving the last vaccine dose, particularly after six months, especially among those aged 60 or older.

**Conclusion:**

The global health challenge of COVID-19 persists, marked by emerging variants with higher virulence and severity, particularly among the unvaccinated and those beyond six months post-vaccination, especially those aged 60 and above. These findings highlight the need for robust surveillance systems targeting new variants and administering booster doses, particularly for individuals aged 60 or older with underlying health conditions, to mitigate the global burden of COVID-19.

**Supplementary Information:**

The online version contains supplementary material available at 10.1007/s44197-024-00298-2.

## Introduction

The Coronavirus Disease 2019 (COVID-19) pandemic has been a public health challenge worldwide due to breakthrough infections and deaths, with the oversaturation of hospitals and intensive care units (ICUs). At the onset of the pandemic, various public health measures, such as mask-wearing, social distancing and lockdowns, were implemented. However, their efficacy was insufficient to curb the rapid spread of the COVID-19 pandemic and mitigate adverse outcomes associated with SARS-CoV-2 infection [1, 2]. Nevertheless, the course of the COVID-19 pandemic changed after the introduction of the SARS-CoV-2 vaccine, whose effectiveness during that time has been widely demonstrated.

One of the primary and most significant risk factors contributing to a higher risk of negative outcomes from COVID-19 infection is age [3]. Thus, despite adequate vaccination coverage, the elderly experienced the highest mortality rates since the beginning of the pandemic [4, 5]. Similarly, patients with chronic diseases such as hypertension, diabetes, cardiovascular disease, or chronic renal failure, as well as those with immunosuppressive conditions like solid or hematological cancer, and HIV infection, promptly exhibited a higher risk of adverse evolution in COVID-19 infection [6, 7]. Consequently, the majority of healthcare systems and governments worldwide adopted similar strategies to prioritize the administration of initial vaccine doses to these high-risk patients, alleviating the substantial health burden caused by COVID-19 and curbing the rapid spread of SARS-CoV-2 infection [8]. The risk profile of individuals with less favorable outcomes from COVID-19 has been the subject of several studies for tailored public health strategies [9, 10]. In this regard, the number of doses administered has been shown to have additive protective effects against adverse outcomes due to SARS-CoV-2 disease, while the duration since administration could result in a reduction of vaccination effectiveness [11–13]. Finally, mutations in the wild variants of COVID-19 have given rise to the development of new variants with increased transmissibility and virulence throughout the evolution of the pandemic. These changes modified the risk profile of individuals in terms of experiencing adverse outcomes from SARS-CoV-2 infection [14] and could impact the effectiveness of current vaccines. Therefore, it is essential to consider the potential emergence of other new variants in the coming years [15]. In this context, it is imperative to implement surveillance systems to monitor and manage any new increases in infection rates, thereby preventing the further spread of COVID-19 [16].

The aim of this study was to assess the risk factors for COVID-19 hospital admission, ICU admission and mortality among the Andalusian population throughout the COVID-19 pandemic (March 2020 to April 2022), and to examine vaccine effectiveness for these severe outcomes in this population.

## Methods

### Study Design and Study Population

We conducted a real-world data region-wide retrospective cohort study in Andalusia, Spain. The Andalusian Health Population Database (BPS) includes demographic and clinical information for all users of the public health system in Andalusia [17]. Each user of the Andalusian public health system is assigned a unique personal identification number, which is used across all primary care centers and hospitals in the region, as well as in all BPS registries.

The study population included all individuals registered in the Andalusian public health system from March 2020 to April 2022. Inclusion criteria required laboratory-confirmed SARS-CoV-2 infection through a validated reverse transcription polymerase chain reaction (RT-PCR) test and sufficient data for analysis. The exclusion criteria were as follows: subjects aged 12 or under (since the vaccination campaign for this age group started later, resulting in a smaller percentage of the population vaccinated); subjects with RT-PCR results registered outside the study period or with incorrect dates; subjects with missing sex information, subjects with missing age data or incorrect data (outliers), subjects who received a vaccine shot before December 27, 2020 (prior to the beginning of the vaccination campaign in Spain); and subjects who received four or more shots of the vaccine during the study period (indicating potential special clinical conditions). The exclusion criteria flowchart, outlining the parameters for exclusion, is referenced in Supplementary Fig. 1.

### SARS-CoV-2 Infection

We defined a SARS-CoV-2 infection in subjects with a positive RT-PCR test during the study period. To characterize SARS-CoV-2 reinfection, we utilized RT-PCR tests conducted within the initial 90 days following the first infection as part of epidemiological surveillance. A minimum time difference of 50 days between the last RT-PCR test and a subsequent positive test was required to establish reinfection.

### SARS-CoV-2 Vaccination

We considered subjects as vaccinated if they had received one, two, or three doses of a COVID-19 vaccine, provided that the last dose was administered at least 14 days before the confirmed laboratory diagnosis of SARS-CoV-2 infection by RT-PCR. The vaccines included in the study were Comirnaty (BioNTech/Pfizer), Spikevax (Moderna), Vaxzevria (AstraZeneca), Jcovden (Janssen), and Nuvaxovid (Novavax). Vaccines not authorized in Europe were excluded from the analysis. The initial phase of the vaccination campaign prioritized older and vulnerable population groups. Subsequently, vaccination efforts gradually expanded to include other age groups. As a result, the date at which each age group achieved 75% vaccine coverage varied. The SARS-CoV-2 vaccination campaign started in the Andalusian region at the end of 2020, with middle-aged adults receiving their first dose by mid-2021. Finally, the booster phase of the SARS-CoV-2 vaccine (third dose) began in October-November 2021. In order to assess the waning effectiveness of the SARS-CoV-2 vaccine and the need for a booster, we categorized the analysis into four groups according to the time since the last vaccine dose was received: less than 30 days ago, 31–90 days ago, 91–180 days ago, and more than 180 days ago.

### Identification of the SARS-CoV-2 Variants

**Fig. 1:**
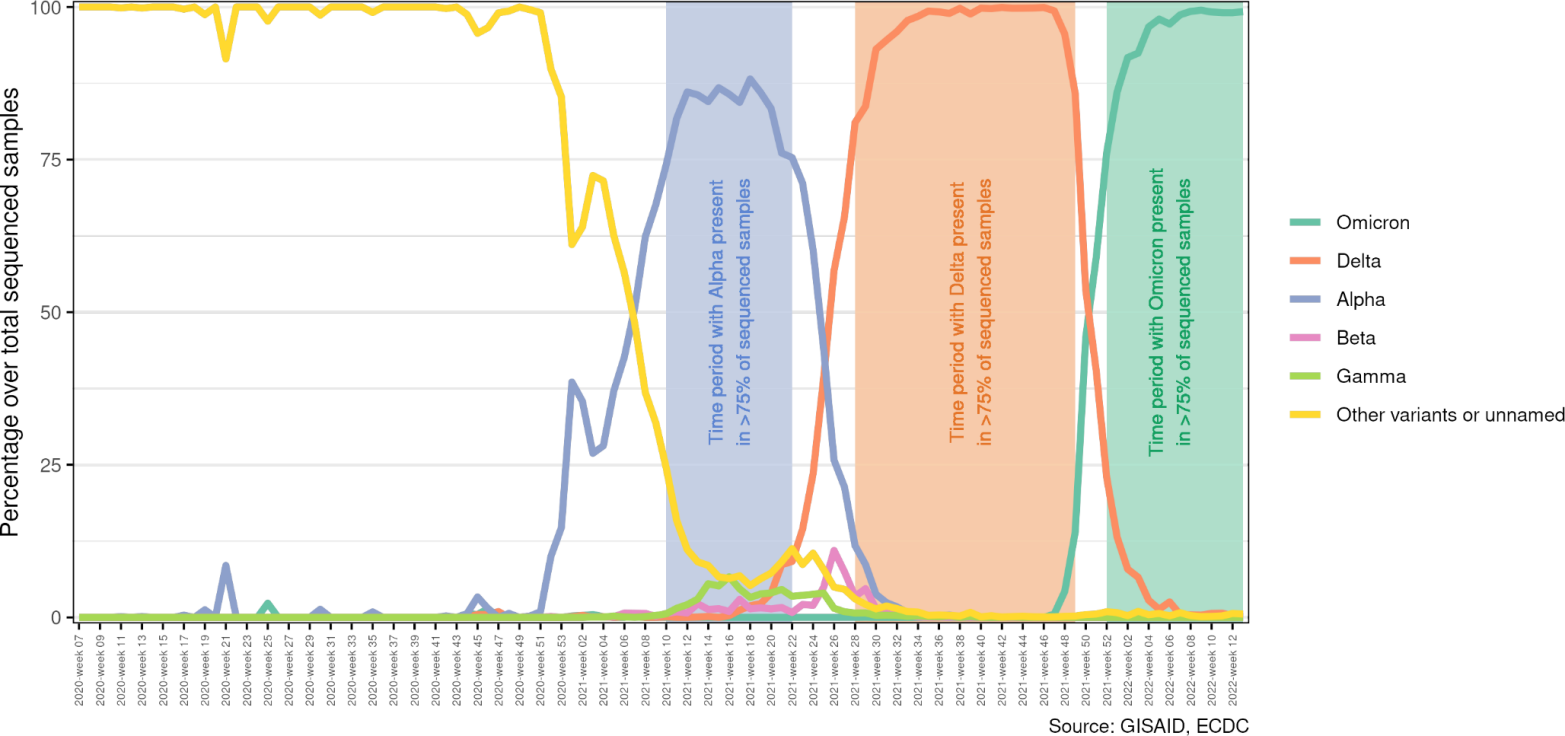
Weekly proportion of SARS-CoV-2 variants sequenced in Spain

The European Centre for Disease Prevention and Control (ECDC) provides weekly updates on COVID-19 sequencing information, including the percentage distribution of variants of concern by week and country. The data from Spain were obtained from the weekly updated downloadable file by ECDC [18], where the variants are referred to by their technical lineage names. To facilitate analysis, their WHO labels were researched and assigned if available. For this purpose, we used information available on the ECDC website [19] and data from the Andalusian circuit for SARS-CoV-2 whole genome sequencing [20]. Considering this information, we defined the period of predominance of a variant of concern as the time when these variants constituted at least 75% of the total distribution observed in laboratory sequencing tests. Therefore, we considered in our analysis four different periods: Before the Alpha variant (other variants) (March 1, 2020, to March 8, 2021), Alpha (March 8 to June 6, 2021), Delta (July 12 to December 12, 2021) and Omicron (December 27, 2021 to March 31, 2022) (Fig. 1).

### Outcomes (Hospital Admission, ICU Admission and Mortality due to SARS-CoV-2 Infection)

Hospitalization data, including admission and discharge dates, ICU admissions, and diagnoses coded according to the International Classification of Diseases 10th version (ICD-10), were extracted from the Andalusia Minimum Basic Data Set (CMBD-A). This dataset was utilized to assess hospital and ICU admissions related to SARS-CoV-2 infection. In this regard, firstly, hospital admission due to COVID-19 was defined as hospitalization within 30 days of confirmed laboratory SARS-CoV-2 infection by RT-PCR and with a diagnostic code associated with SARS-CoV-2 infection among the first five discharge diagnoses codes for the patient (Supplementary Table 1). Secondly, ICU admission due to COVID-19 was considered if the patient was admitted to the ICU within 30 days of confirmed SARS-CoV-2 infection by RT-PCR during hospitalization, as previously defined. Thirdly, mortality due to COVID-19 was defined as death occurring within 30 days of confirmed laboratory SARS-CoV-2 infection by RT-PCR during the hospitalization for SARS-CoV-2 infection. Additionally, ambulatory death due to COVID-19 was considered if the patient died within 30 days of confirmed laboratory SARS-CoV-2 infection by RT-PCR.

### Covariates

According to previous studies, a number of covariates have been considered in this work [21, 22]. Information regarding age, sex (male/female), and the presence of chronic diseases at the time when the patient had a confirmed laboratory SARS-CoV-2 infection were included in the analysis. The considered chronic diseases encompassed asthma, hypertension, type 2 mellitus diabetes, chronic obstructive pulmonary disease (COPD), ischemic heart disease, chronic renal disease, heart failure disease, human immunodeficiency virus-positive (HIV+), chronic hepatic disease (except cirrhosis), hepatic cirrhosis, active solid cancer (including lung, head and neck, gastric, bone, soft or connective tissues, breast, ovarian, pancreatic, prostate, renal, thyroid, uterine, bladder and melanoma) and hematological cancer (including immunoproliferative cancer, Hodgkin lymphoma, non-Hodgkin lymphoma, leukemia) diagnosed within the last 5 years.

### Statistical Analysis

Generalized Additive Model (GAM) technique emerges as a powerful tool that combines the robustness and reliability of complex machine learning models with the distinctive advantage of producing interpretable and intuitive outcomes, akin to what classic logistic regressions offer [23]. Our primary objective was to understand the nuanced interplay between the three main binary response variables (hospital admission, ICU admission and death), and a diverse array of predictor variables, which included demographic and health-related attributes. In the process of constructing the GAM model, we adopted a strategic approach that involved fitting smooth and flexible non-linear relationships to relevant variables. Particularly, the association between age and the binary outcome was captured using a smooth function with the consideration of four knots. This methodological decision was instrumental in capturing potential non-linear associations. In this regard, the study explored the non-linear relationship between age and the three specified dependent outcome variables.

To analyze vaccine effectiveness based on the number of vaccine doses, we categorized cases into periods characterized by the predominance of variants, distinguishing between individuals under 60 years old and those above 60 years old. We considered the doses of SARS-CoV-2 vaccine received according to the vaccination campaign. The categorized periods included: before the Alpha variant period for individuals above 60 years old receiving one dose; during the Alpha variant period for individuals above 60 years old receiving one and two doses; during the Delta variant period for individuals under 60 years old receiving one and two doses; during the Delta variant period for individuals above 60 years old receiving one, two, and three doses; and during the Omega variant period for individuals under 60 years old and above 60 years old receiving one, two, and three doses. We considered the age group vaccinated after reaching 75% vaccine coverage. Finally, we conducted an analysis to assess the risk of severe outcomes based on the time since the last vaccine dose, comparing the first 30 days after administration with the following intervals: 31–90 days, 91–180 days, and more than 180 days.

To mitigate potential sources of bias, rigorous data cleansing techniques were employed during the study. These included incorporating clinical expertise, validating data thoroughly, and ensuring the accuracy of demographic and clinical information in the Andalusian Health Population Database. The methodology focused on measures like addressing missing data (e.g., sex, age), verifying COVID-19 vaccine administration timing, scrutinizing hospitalization records, and excluding cases with unusual vaccine dosages. These steps aimed to enhance the precision and reliability of the study’s outcomes.

The analyses were performed using R software version 4.0.3 (R Foundation for Statistical Computing, Vienna, Austria). The packages lubridate, dtplyr, data.table, vroom, tableone, mgcv and those included in the tidyverse were employed to improve overall performance, conduct statistical analyses and plot figures. The authors provide open access to all their data wrangling, filtering, modeling, figures and tables code [24].

## Results

We included a total of 686,519 cases of SARS-CoV-2 infection from 671,229 subjects from March 2020 to April 2022, after applying exclusion criteria previously described in Supplementary Fig. 1. The baseline characteristics of the cases of laboratory SARS-CoV-2 infection according to the different outcomes analyzed (hospital admission, ICU admission, and 30-day mortality) are shown in Table 1.

**Table 1:** Baseline characteristics of cases of confirmed laboratory SARS-CoV-2 infection by reverse transcription polymerase chain reaction (RT-PCR)

*n* (%)	Total: 686,519 cases	Hospital stay		*p* ^c^	ICU stay		*p* ^c^	30-day mortality		*p* ^c^
		No: 638,323	Yes: 48,196		No: 43,139	Yes: 5,057		No: 675,230	Yes: 11,289	
Age (years)^a^	43.15 (19.37)	41.61 (18.61)	63.54 (17.60)	< 0.01	64.06 (17.99)	59.11 (13.01)	< 0.01	42.57 (18.94)	77.59 (12.60)	< 0.01
Hospital stay duration (days)^b^		-	8 (5, 14)	NA	8 (5, 12)	21 (14, 36)	< 0.01	-	-	NA
ICU stay duration (days)^b^		-	-	NA	-	11 (5, 22)	NA	-	-	NA
Sex (male)	306,903 (45%)	279,491 (43.8%)	27,412 (56.9%)	< 0.01	23,914 (55.4%)	3,498 (69.2%)	< 0.01	300,709 (44.5%)	6,194 (54.9%)	< 0.01
Ashtma	86,920 (13%)	81,422 (12.8%)	5,498 (11.4%)	< 0.01	4,999 (11.6%)	499 (9.9%)	< 0.01	85,889 (12.7%)	1,031 (9.1%)	< 0.01
COPD	32,924 (4.8%)	25,396 (4%)	7,528 (15.6%)	< 0.01	6,973 (16.6%)	555 (11%)	< 0.01	30,213 (4.5%)	2,711 (24%)	< 0.01
Hypertension	162,322 (24%)	134,549 (21.1%)	27,773 (57.6%)	< 0.01	25,057 (58%)	2,716 (53.7%)	< 0.01	153,661 (22.8%)	8,661 (76.7%)	< 0.01
Ischemic heart disease	18,389 (2.7%)	13,616 (2.1%)	4,773 (9.9%)	< 0.01	4,357 (10.1%)	416 (8.2%)	< 0.01	16,546 (2.5%)	1,843 (16.3%)	< 0.01
Heart failure	23,484 (3.4%)	15,919 (2.5%)	7,565 (15.7%)	< 0.01	7,186 (16.7%)	379 (7.5%)	< 0.01	20,094 (3%)	3,390 (30%)	< 0.01
Diabetes	68,242 (9.9%)	53,734 (8.4%)	14,508 (30%)	< 0.01	12,978 (30%)	1,530 (30.2%)	0.82	63,493 (9.4%)	4,749 (42%)	< 0.01
Hepatic cirrhosis	2,523 (0.4%)	1,922 (0.3%)	601 (1.2%)	< 0.01	542 (1.3%)	59 (1.2%)	0.63	2,244 (0.3%)	279 (2.5%)	< 0.01
Chronic hepatic disease (except cirrhosis)	13,722 (2.0%)	11,266 (1.8%)	2,456 (5.1%)	< 0.01	2,190 (5.1%)	266 (5.3%)	0.60	12,939 (1.9%)	783 (6.9%)	< 0.01
Chronic kidney disease	16,460 (2.4%)	10,993 (1.7%)	5,467 (11.3%)	< 0.01	5,105 (11.8%)	362 (7.2%)	< 0.01	14,028 (2.1%)	2,432 (21.5%)	< 0.01
HIV+	1,924 (0.3%)	1,708 (0.3%)	216 (0.4%)	< 0.01	197 (0.5%)	19 (0.4%)	0.48	1,890 (0.3%)	34 (0.3%)	0.74
Solid organ cancer	30,585 (4.5%)	25,149 (3.9%)	5,436 (11.3%)	< 0.01	5,049 (11.7%)	387 (7.7%)	< 0.01	28,535 (4.2%)	2,050 (18.2%)	< 0.01
Hematologic cancer	3,783 (0.6%)	2,663 (0.4%)	1,120 (2.3%)	< 0.01	984 (2.3%)	136 (2.7%)	0.08	3,393 (0.5%)	390 (3.5%)	< 0.01
COVID vaccination (doses)										
*None*	406,186 (59%)	369,300 (57.9%)	36,886 (76.5%)	< 0.01	32,611 (75.6%)	4,275 (84.5%)	< 0.01	398,275 (59%)	7,911 (70.1%)	< 0.01
*One*	36,026 (5.2%)	34,277 (5.4%)	1,749 (3.6%)	< 0.01	1,566 (3.6%)	183 (3.6%)	< 0.01	35,692 (5.3%)	334 (3%)	< 0.01
*Two*	180,411 (26%)	174,505 (27.3%)	5,906 (12.2%)	< 0.01	5,474 (12.7%)	432 (8.5%)	< 0.01	178,863 (26.5%)	1,548 (13.7%)	< 0.01
*Three*	63,896 (9.3%)	60,241 (9.4%)	3,655 (7.6%)	< 0.01	3,488 (8.1%)	167 (3.3%)	< 0.01	62,400 (9.2%)	1,496 (13.3%)	< 0.01
Previous COVID infections (count)										
*None*	671,229 (98%)	623,493 (97.7%)	47,736 (99%)	< 0.01	42,710 (99%)	5,026 (99.4%)	0.03	660,087 (97.8%)	11,142 (98.7%)	< 0.01
*One*	15,120 (2.2%)	14,665 (2.3%)	455 (0.9%)	< 0.01	424 (0.9%)	31 (0.6%)	0.03	14,975 (2.2%)	145 (1.3%)	< 0.01
*Two*	170 (< 0.1%)	165 (0.03%)	5 (0.01%)		5 (0.01%)	0 (0%)		168 (0.02%)	2 (0.02%)	
Time since last immunisation										
*No immunisation*	402,891 (59%)	366,143 (57.4%)	36,748 (76.2%)	< 0.01	32,486 (75.3%)	4,262 (84.3%)		395,024 (58.5%)	7,867 (69.7%)	< 0.01
*Less than one month*	30,387 (4.4%)	29,169 (4.6%)	1,218 (2.5%)	< 0.01	1,114 (2.6%)	104 (2.1%)		30,093 (4.5%)	294 (2.6%)	< 0.01
*One to three months*	68,118 (9.9%)	65,059 (10.2%)	3,059 (6.3%)	< 0.01	2,821 (6.5%)	238 (4.7%)		67,207 (10%)	911 (8.1%)	< 0.01
*Three to six months*	110,591 (16%)	106,319 (16.7%)	4,272 (8.7%)	< 0.01	4,022 (9.3%)	250 (4.9%)		109,116 (16.2%)	1,475 (13.1%)	< 0.01
*Six months or more*	74,532 (11%)	71,633 (11.2%)	2,899 (6%)	< 0.01	2,696 (6.2%)	203 (4%)		73,790 (10.9%)	742 (6.6%)	< 0.01
Variant period										
*αpredominance*	56,380 (8.2%)	50,385 (7.9%)	5,995 (12.4%)	< 0.01	5,318 (12.3%)	677 (13.4%)	< 0.01	55,712 (8.3%)	668 (5.9%)	< 0.01
*Δpredominance*	102,986 (15%)	97,282 (15.2%)	5,704 (11.8%)	< 0.01	5,050 (11.7%)	654 (12.9%)	< 0.01	101,832 (15.1%)	1,154 (10.2%)	< 0.01
*Ό predominance*	185,357 (27%)	177,895 (15.2%)	7,462 (15.5%)	< 0.01	6,955 (16.1%)	507 (10%)	< 0.01	183,127 (27.1%)	2,230 (19.8%)	< 0.01

^a^ Expressed as mean and standard deviation. ^b^ Expressed as median and interquartile range. ^c^ Wilcoxon test for quantitative variables, chi-squared test for categorical variables

Abbreviations: COPD, chronic obstructive pulmonary disease; COVID, coronavirus disease; HIV, human immunodeficiency virus; ICU, intensive care unit; NA, not applicable

In summary, the study included 48,196 cases of hospital admission, 5,057 cases of ICU admission and 11,289 deaths due to SARS-CoV-2 infection (Supplementary Figs. 2–4). Individuals who were older, male, and had a higher prevalence of chronic diseases were more likely to be admitted to the hospital and expire due to SARS-CoV-2 infection. However, COVID-19 vaccination doses contributed to a reduction in COVID-19-related hospital admissions, ICU admissions and deaths. Moreover, the booster dose of the vaccine further enhanced this reduction.

### Risk Profile

**Fig. 2:**
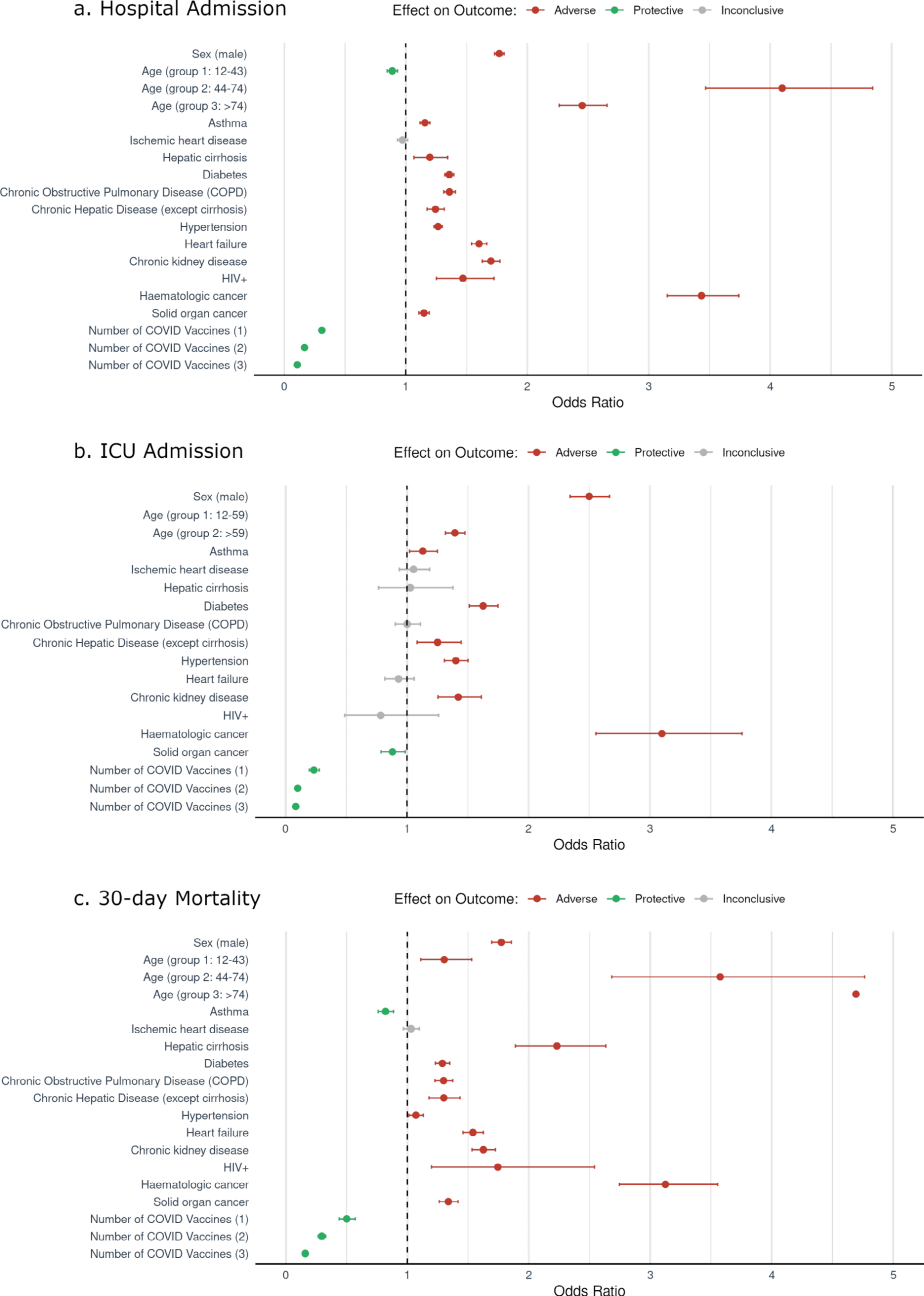
Odds ratios and confidence intervals for (**a**) Hospital admission (**b**) ICU admission (**c**) 30-day mortality associated factors due to COVID-19 infection

Male sex was significantly associated with hospital admission (OR 1.77 (95% CI 1.73–1.81, *p* < 0.01)), ICU admission (OR 2.50 (95% CI 2.34–2.67, *p* < 0.01)), and mortality (OR 1.77 (95% CI 1.69–1.85, *p* < 0.01)). Hematologic cancer showed a strong association with hospital admission (OR 3.43 (95% CI 3.15–3.74, *p* < 0.01)), ICU admission (OR 3.10 (95% CI 2.55–3.76, *p* < 0.01)), and mortality (OR 3.12 (95% CI 2.75–3.55, *p* < 0.01)). Additionally, age emerged as a significant factor for all three outcomes (Fig. 2).

**Fig. 3:**
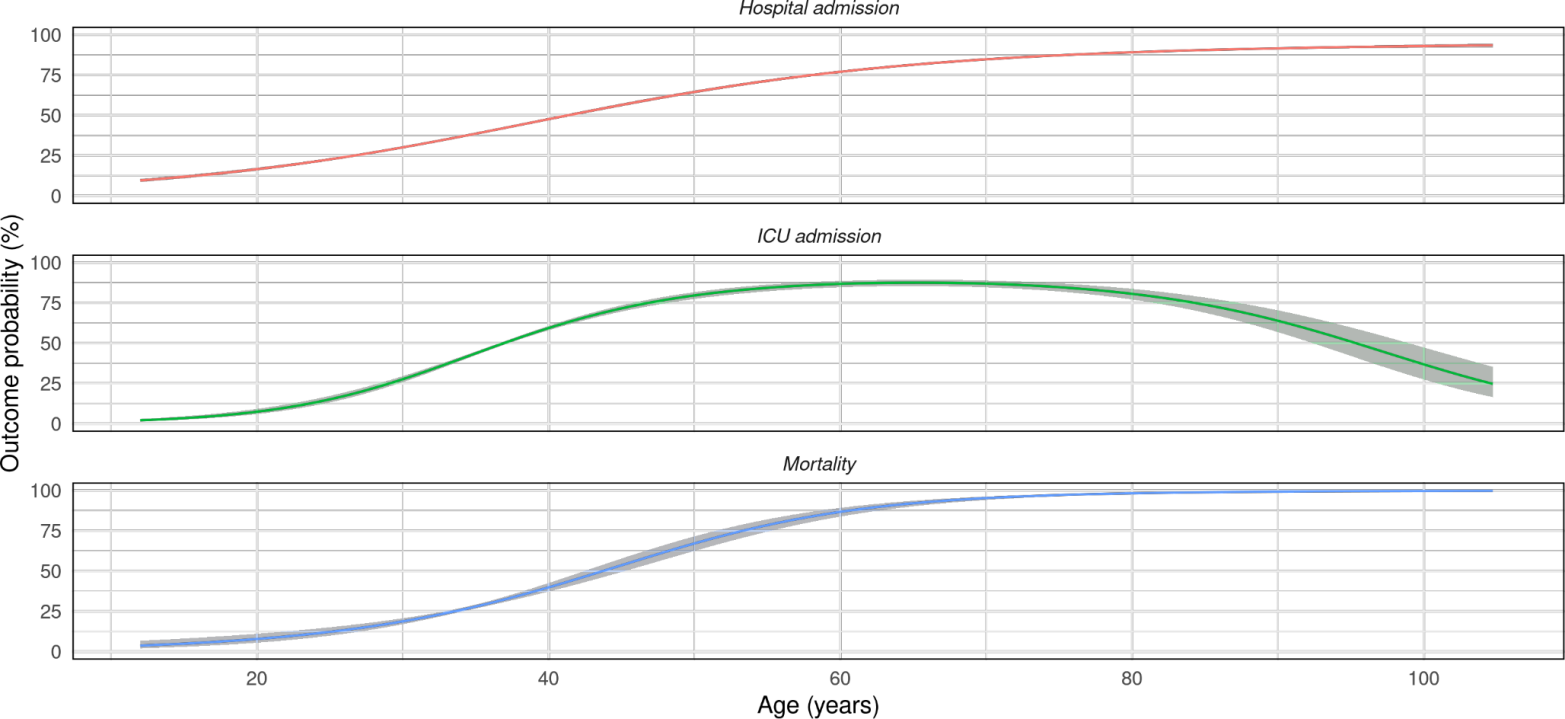
Smooth and flexible non-linear associations between age and the risk for hospital admission, ICU admission and mortality due to COVID-19 infection

More specifically, age shows a non-linear relationship with the three outcomes: older patients have a lower probability of ICU admission, while the probability of hospital admission and mortality progressively increases with age (Fig. 3).

In this regard, the age group 44–74 years showed a higher odds ratio (OR 4.10 (95% CI 3.47–4.84, *p* < 0.01)) than the age group > 74 years (OR 2.45 (95% CI 2.26–2.66, *p* < 0.01)) for hospital admission. Conversely, for mortality, the age group > 74 years had an OR of 4.69 (95% CI 3.77–5.84, *p* < 0.01), while the age group 44–74 years had an OR of 3.57 (95% CI 2.68–4.76, *p* < 0.01). Asthma is considered an adverse factor associated with hospital admission (OR 1.16 (95% CI 1.12–1.20, *p* < 0.01)); however, it was a protective factor for mortality (OR 0.82 (95% CI 0.76–0.89, *p* < 0.01)). For ICU admission, certain chronic diseases that were considered adverse factors for hospital admission and mortality, such as hepatic cirrhosis, COPD, heart failure, and HIV, appeared as inconclusive factors. Surprisingly, solid organ cancer is even identified as a protective factor (OR 0.88 (95% CI 0.79–0.99, *p* < 0.01)). Ischemic heart disease was identified as an inconclusive associated factor in all three outcomes. Diabetes, hypertension, chronic hepatic disease and chronic kidney disease were identified as adverse associated factors for the three outcomes.

The comparative analyses of odds ratios before the vaccination introduction and after achieving 75% vaccine coverage indicated that, following this coverage, the oldest patients (> 74 years) had higher odds for mortality (OR 5.67 (95% CI 4.05–7.93, *p* < 0.01)), but lower odds for hospital and ICU admission compared to the other age groups. Before vaccination, the age group of 44–74 years had higher odds for mortality (OR 4.96 (95% CI 3.25–7.59, *p* < 0.01)) than the age group > 74 years. Almost all chronic diseases exhibited a reduction in the odds of mortality once vaccination coverage reached 75%. However, they demonstrated an increase in the likelihood of hospital admission after reaching the same coverage threshold (Supplementary Fig. 5).

### Vaccine Effectiveness

The vaccine emerged as a protective factor associated with all three outcomes, as the odds of experiencing any of them decreased with the number of COVID-19 vaccine doses. Notably, three doses of COVID vaccines displayed the most protective effect, with an OR of 0.11 (95% CI 0.10–0.11, *p* < 0.01) for hospital admission, an OR of 0.08 (95% CI 0.07–0.10, *p* < 0.01) for ICU admission, and an OR of 0.16 (95% CI 0.14–0.18, *p* < 0.01) for mortality (Fig. 2).

Further analyses were conducted to evaluate vaccine effectiveness concerning the period of variant predominance, considering age and the number of COVID vaccine doses. The results indicated a positive correlation between vaccine effectiveness and the number of COVID vaccine doses for all three outcomes (hospital admission, ICU admission, and mortality), particularly among individuals aged 60 years or older (Supplementary Figs. 6–8). Interestingly, during the Omicron variant, patients required three vaccine doses to achieve a vaccine effectiveness similar to that observed with other variants.

### Time of Vaccine Effectiveness

**Fig. 4:**
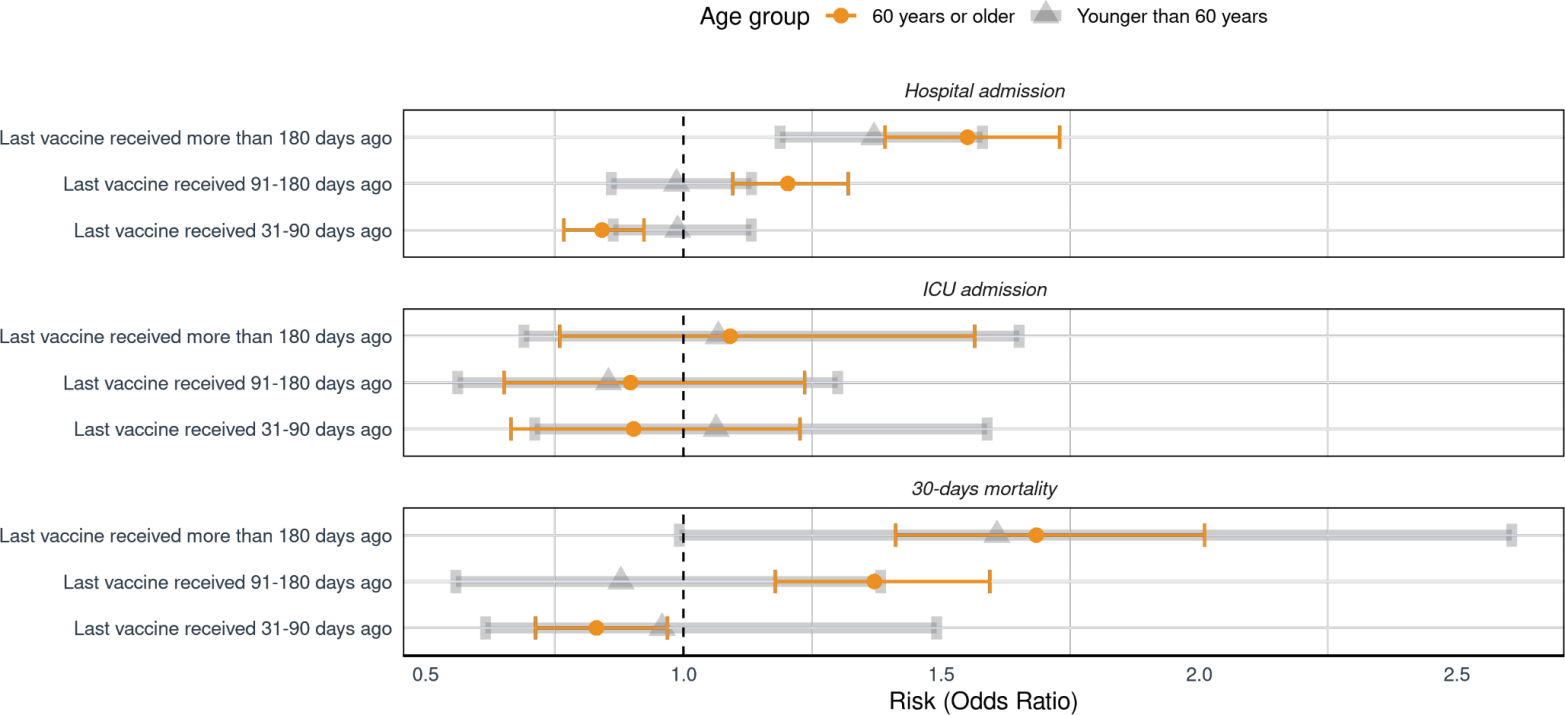
Risk of hospital admission, ICU admission and mortality due to COVID-19 infection, categorized by age (60 years or older and younger than 60 years) and time elapsed since the last vaccine received

The analyses assessing the duration of vaccine effectiveness revealed a waning protective effect for hospital admission after vaccination, especially among individuals aged 60 years or older. The OR were 1.20 (95% CI 1.10–1.32, *p* < 0.01) for individuals whose last vaccine was received 91–180 days ago and 1.55 (95% CI 1.39–1.73, *p* < 0.01) for those whose last vaccine was received more than 180 days ago, compared to those who received their last vaccine less than 30 days ago. Likewise, the protective effect among the population aged less than 60 years demonstrated signs of waning over time, revealing an OR of 1.37 (95% CI 1.19–1.58, *p* < 0.01) for hospital admission when the last vaccine was administered more than 180 days ago, in comparison to those who received the last vaccine less than 30 days ago. Furthermore, our findings indicated a waning protective effect against mortality in the population aged 60 years or older as time elapsed since receiving the vaccine. Specifically, individuals with their last vaccine received 91–180 days ago exhibited an odds ratio of 1.37 (95% CI 1.18–1.59, *p* < 0.01), while those with their last vaccine received more than 180 days ago had an odds ratio of 1.68 (95% CI 1.41–2.01, *p* < 0.01) (Fig. 4).

## Discussion

Our study provides evidence about the effectiveness of the SARS-CoV-2 vaccine and the booster of the vaccine against hospital admission, ICU admission, and mortality associated with COVID-19, as well as the impact of pre-existing comorbidities as potential risk factors influencing the progression of COVID-19 infection. Our study shows that age, male sex, hematologic cancer, hypertension, chronic hepatic disease, chronic kidney disease and mellitus diabetes are risk factors for hospital admission, ICU admission, and mortality due to COVID-19 infection. During the omicron period, the effectiveness of the vaccine, particularly among individuals aged 60 years and older, was lower compared to the previous period dominated by other variants (before alpha, alpha and delta periods). Hence, our findings indicate a heightened risk of hospital admission and mortality due to COVID-19 infection with increasing time since vaccine exposure, particularly among individuals aged 60 years or older. This highlights the need for administering booster doses of the SARS-CoV-2 vaccine.

Previous studies have shown that age, male sex, chronic diseases and immunosuppressive conditions, such as cancer, contribute to an increased risk of experiencing a more severe course of illness from SARS-CoV-2 infection [25–27]. However, our study revealed a non-linear relationship with age as a risk factor and the analyzed outcomes. There was a progression in risk at middle ages, while the risk for both younger and older individuals remains relatively stable. Nevertheless, the risk of ICU admission did decrease in older individuals. Interestingly, age groups of more than 74 years showed lower odds of suffering hospital and ICU admission than the age group between 44 and 74 years. These findings highlight the effectiveness of health policy measures implemented during the COVID-19 pandemic in the Andalusian region, ensuring nursing homes had sufficient medical equipment and offering primary care clinicians direct and continuous support through ongoing communication with hospital services, thus preventing hospital admissions among older patients with mild to moderate COVID-19 infections.

In our study, hematological cancer has shown the highest risk to suffer worse evolution due to COVID-19 infection. Similarly, prior studies by Pagano L. et al. and Song Q. have previously demonstrated a higher likelihood of worsening outcomes for patients with hematological cancers compared to other types of cancer and the general population, particularly in the context of COVID-19 [28, 29]. In fact, the response after vaccination against SARS-CoV-2 in adults with hematological cancer have been demonstrated to be lower compared to adults with solid cancer or healthy adults, with the administered treatment identified as the main influencing factor [30]. The results shown for asthma in previous studies are inconclusive in determining whether it should be considered a risk or protective factor for hospital admission and mortality due to SARS-CoV-2. The findings from two meta-analysis assessing the risk for mortality among patients with COVID-19 in the United Kingdom (1,209,675 COVID-19 patients) and United States (426,261 COVID-19 patients) demonstrated that pre-existing asthma was associated with reduced risk for COVID-19 mortality [31, 32]. However, in another meta-analysis involving a total of 1,229,434 COVID-19 patients, the odds ratio for mortality associated with pre-existing asthma was inconclusive (OR 0.89 (95% CI 0.55–1.4; *p* = 0.630)) [33]. Therefore, additional studies assessing the severity of asthma, the efficacy of treatments employed for its management, and their correlation with mortality among COVID-19 patients, are needed to clarify these unclear findings. Additionally, it is worth noting that previous studies have suggested a potential protective effect associated with the use of inhaled corticosteroids [34, 35].

The key role of SARS-CoV-2 vaccines in the COVID pandemic against COVID-19 infection and severe outcomes has been widely demonstrated. Initially shown in the clinical trials of leading mRNA vaccines, their safety and efficacy were prominently established. Subsequent real-world data studies further affirmed these findings, reinforcing the vital contribution of these vaccines to the ongoing battle against COVID-19 [36, 37]. In addition, the waning of the vaccine effectiveness over time has been demonstrated by Nordström P. et al. supporting the administration of a third vaccine dose as a booster [38]. In fact, the clinical trial demonstrated the safety and effectiveness of a booster vaccine against COVID-19, and meta-analyses of longitudinal studies have demonstrated the effectiveness of this booster compared to previous full vaccination (2 doses administered) against severe outcomes due to COVID-19 [39–42]. Our findings consistently indicated lower odds of hospital admission, ICU admission, and mortality associated with COVID-19 among individuals who received three vaccine doses. This trend held true across general analyses and was further reinforced in sub-analyses, where we observed heightened vaccine effectiveness for individuals with three doses, particularly during the Omicron variant period. Several rigorous studies have addressed the decline in vaccine effectiveness over time following its administration. Our study aligns with and supports these findings, particularly emphasizing the importance of administering booster doses of the SARS-CoV-2 vaccine. These collective insights serve as a foundation for shaping future public health measures and informing targeted vaccination campaigns, with a focus on extending coverage to the more vulnerable populations [43, 44]. In fact, Andrews N. et al. demonstrated that the vaccine effectiveness waning was greater in older adults and individuals in a clinical risk group [45]. Our findings revealed a progressive increase in risk over time following the administration of the vaccine dose within the Andalusian population with COVID-19. This increase was particularly pronounced among individuals aged 60 years or older, where COVID-19 patients exhibited higher odds of hospital admission and mortality six months post-vaccine administration. These results support the need for booster vaccines, especially among older patients with comorbidities.

Finally, the appearance of different SARS-CoV-2 variants of concern during the COVID-19 pandemic has been the focus of attention. This focus is attributed to the fact that new mutations tend to induce increased virulence, severity, and resistance to both natural infection and vaccine-induced immunity [46, 47]. The last variant of concern, Omicron, which emerged in November 2021, has been demonstrated to be more contagious than previous variants with higher rates of infection worldwide [48, 49]. The efficacy of the vaccine on the Omicron variant was questioned due to the time since the last vaccine was administered and a booster shot of the vaccine started to be administered. However, previous studies have demonstrated the efficacy of the booster vaccine in reducing the hospital admission, ICU admission and mortality by Omicron variant, especially when compared to individuals who are unvaccinated or have received fewer vaccine doses [50, 51]. Similarly, our results indicated that a booster was necessary to achieve comparable vaccine effectiveness in reducing hospital admission, ICU admission, and mortality during the Omicron period. Individuals with only one or two doses of the vaccine exhibited lower effectiveness.

Several limitations should be noted in the context of this study. While RT-PCR was initially used as the main diagnostic test for SARS-CoV-2 infection, antigen-detection rapid diagnostic tests (Ag-RDTs) in nasal swabs were developed in September 2020. Consequently, the utilization of Ag-RDTs for early identification of infections and prevention of their spread increased steadily throughout the study period. In this study, only subjects with laboratory confirmed SARS-CoV-2 infection by RT-PCR were included; therefore, there might be an underrepresentation of mild COVID-19 cases diagnosed with Ag-RDTs, potentially leading to an overestimation of the results obtained in our study. Nevertheless, throughout the study period, the recommended protocol involved verifying a positive rapid antigen test in an ambulatory setting through RT-PCR confirmation. In this regard, upon hospital admission, a laboratory test using RT-PCR was performed. For this reason, due to the lack of specific symptom onset information in the dataset, we chose not to use the time from diagnostic test to hospital admission as an indicator of disease progression in our models. This variable did not accurately reflect the onset of COVID-19 and may be unreliable, as there were cases where RT-PCR testing was conducted after hospital admission. Furthermore, within the Andalusian digital healthcare system, certain risk factors that have evidence of contributing to a worsened prognosis in COVID-19 infection, such as obesity or smoking status, are not comprehensively recorded. As a result, these factors were not included in the analyses. For patients with a history of cancer, the stage of the disease was unavailable for inclusion in the analyses, even though it could have a direct impact on the prognosis of COVID-19 infection. We conducted various analyses to assess the impact of vaccines and risk factors during periods of the predominant SARS-CoV-2 variant. However, waves during the COVID-19 pandemic were not considered in our study. It is crucial to note that the oversaturation of hospitals and ICUs during different waves could have influenced the admission criteria in Andalusian healthcare facilities. 

On the other hand, this study has important strengths. Firstly, it unveils real-world evidence derived from an extensive dataset encompassing over 9 million patients, making the patient sample representative of the total Andalusian region. Secondly, the 2-year study duration enables a thorough examination of patient evolution, SARS-CoV-2 variants of concern, and vaccine effectiveness from the onset of the pandemic. Thirdly, employing GAM as a machine learning model facilitated the exploration of the non-linear relationship between age and hospital admission, ICU admission, and mortality. Ultimately, our study demonstrates the effective management of the vaccination campaign in Andalusia, Spain, showcasing its positive impact on public health.

## Conclusion

In conclusion, the vaccine campaigns and public health policies have proven effective in controlling the COVID-19 pandemic among the Andalusian population. Male patients above 60 years old with comorbidities, especially diabetes, hypertension, chronic hepatic and kidney disease, and hematological cancer, have been shown to be more vulnerable to hospital admission, ICU admission and mortality due to COVID-19. These findings should be taken into account for future public health policy measures. Furthermore, the efficacy of the vaccine diminishes over time following the last administered dose, highlighting the importance of administering booster doses of the SARS-CoV-2 vaccine to mitigate hospital admissions, ICU admissions, and deaths.

Real-world data studies based on electronic health records (EHRs) present valuable opportunities for analyzing and tailoring public health measures. These studies highlight the vital role of responsibly using data to improve public well-being, particularly in addressing health challenges like the COVID-19 pandemic. This supports decision-makers in their efforts. Given these insights, it becomes imperative to draw lessons from the findings and develop strategies aimed at mitigating risks for preventing and managing potential COVID outbreaks and crises. The relevance of these findings extends to stakeholders in global health, especially those involved in policy and clinical decision making.

## Electronic Supplementary Material

Below is the link to the electronic supplementary material.

Supplementary Material 1

## Data Availability

Data are available upon reasonable request.

## Abbreviations

Ag-RDT: Antigen-detection rapid diagnostic test
BPS: Andalusian health population database
CI: Confidence interval
CMBD-A: Andalusia minimum basic data set
COPD: Chronic obstructive pulmonary disease
COVID-19: Coronavirus disease 2019
ECDC: European Centre for Disease Prevention and Control
EHR: Electronic health record
Fig: Figure
GAM: Generalized additive model
HIV: Human immunodeficiency virus
ICD-10: International Classification of Diseases 10th version
ICU: Intensive care unit
NA: Non applicable
OR: odds ratio
P: p-value
RT-PCR: Reverse transcription polymerase chain reaction
SARS-CoV-2: Severe acute respiratory syndrome coronavirus 2
WHO: World Health Organization

## Code Availability

The authors provide open access to all their data wrangling, filtering, modeling, figures and tables code in https://github.com/theonesp/vacunas_primario/tree/main.
