# Supplementary material for: Assessing COVID-19 Vaccine Effectiveness and Risk Factors for Severe Outcomes through Machine Learning Techniques: A Real-World Data Study in Andalusia, Spain
Source: J Epidemiol Glob Health. 2024 Nov 11;14(4):1504–17. doi: 10.1007/s44197-024-00298-2 (PMC11652453; doi:10.1007/s44197-024-00298-2)
Supplement: Supplementary file 1 — Supplementary Material 1 [file 44197_2024_298_MOESM1_ESM.pdf]

Supplementary Material

Assessing COVID-19 Vaccine Effectiveness and Risk Factors for Severe Outcomes through Machine Learning Techniques: A Real-World Data Study in Andalusia, Spain

Journal of Epidemiology and Global Health

Álvaro Serrano-Ortiz[# 1,2,3], Juan Luis Romero-Cabrera[# 4,5], Jaime Monserrat Villatoro[6,7], Jaime Cordero-Ramos[8,9,10], Rafael Ruiz-Montero[1,2,11], Álvaro Ritoré[12], Joaquín Dopazo[10,13], Jorge del Diego Salas[14], Valle García Sánchez[15,16], Inmaculada Salcedo-Leal[¶1,2,11], Miguel Ángel Armengol de la Hoz [¶12], Isaac Túnez[¶7,16,17,18], Miguel Ángel Guzmán[¶19]*

# Co-first author, Contributed equally.

¶ Co-senior author, Contributed equally.

** Corresponding author. Email: mangel.armengol@juntadeandalucia.es*

[1]Preventive Medicine and Public Health Unit, Reina Sofía University Hospital, Córdoba, Spain

[2]Preventive Medicine and Public Health Research Group, Maimonides Biomedical Research Institute of Córdoba (IMIBIC), Córdoba, Spain.

[3]Preventive Medicine and Public Health Unit, Healthcare Management Area: South of Córdoba, Cabra, Córdoba, Spain

[4]Lipids and Atherosclerosis Unit, Maimonides Biomedical Research Institute of Córdoba (IMIBIC), Reina Sofia University Hospital, University of Córdoba, Córdoba, Spain

[5]CIBEROBN (CIBER in Physiopathology of Obesity and Nutrition), Instituto de Salud Carlos III, Madrid, Spain

[6]Health District of Córdoba and Guadalquivir, Córdoba, Spain

[7]Maimonides Biomedical Research Institute of Córdoba (IMIBIC), Córdoba, Spain

[8]Pharmaceutical Management Department, Extremadura Health Service, Mérida, Spain

[9]Hospital Pharmacy, Virgen Macarena University Hospital, Seville, Spain

[10]Institute of Biomedicine of Seville (IBiS)/University Hospital Virgen del Rocío/CSIC/University of Sevilla, Seville, Spain

[11]Department of Medical and Surgical Sciences, University of Córdoba, Córdoba, Spain

[12]Big Data Department, PMC-FPS, Regional Ministry of Health and Consumer Affairs, Seville, Spain

[13]Computational Medicine Platform, Andalusian Public Foundation Progress and Health-FPS, Seville, Spain

[14]Directorate General of Public Health and Pharmaceutical Regulation, Ministry of Health and Consumer Affairs of the Regional Government of Andalusia, Seville, Spain

[15]Management Directorate of Andalusian Health Service, Ministry of Health and Consumer Affairs of the Regional Government of Andalusia, Seville, Spain

[16]Reina Sofía University Hospital, Córdoba, Spain

[17]Department of Biochemistry and Molecular Biology, University of Córdoba, Córdoba, Spain

[18]General Secretariat of Public Health and Research, Development and Innovation in Health, Ministry of Health and Consumer Affairs of the Regional Government of Andalusia, Seville, Spain

[19]Andalusian Public Healthcare System, Andalusia, Spain

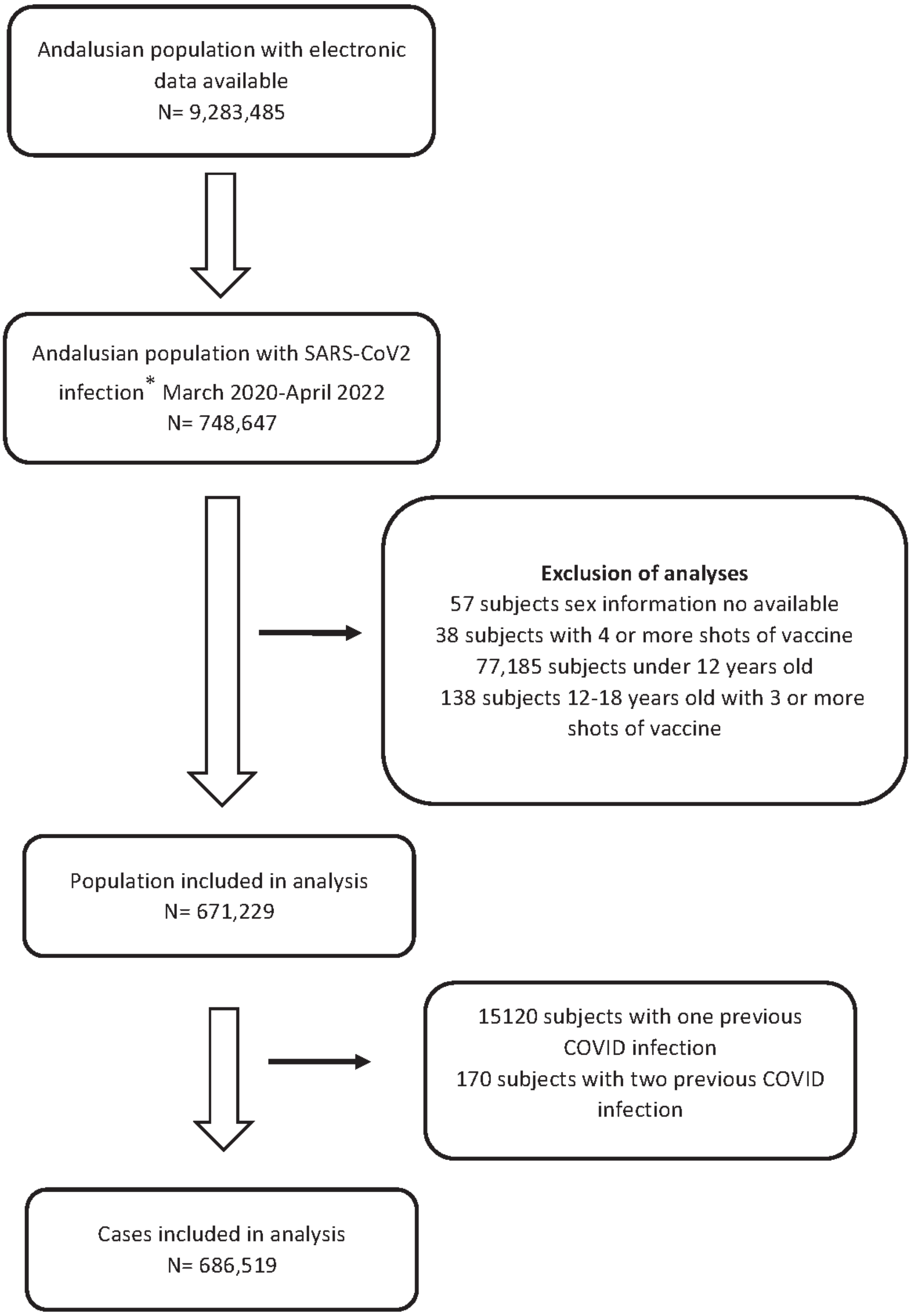

Supplementary Fig. 1 Flowchart of the population included in the current analysis

Sample distribution flows towards hospital admission

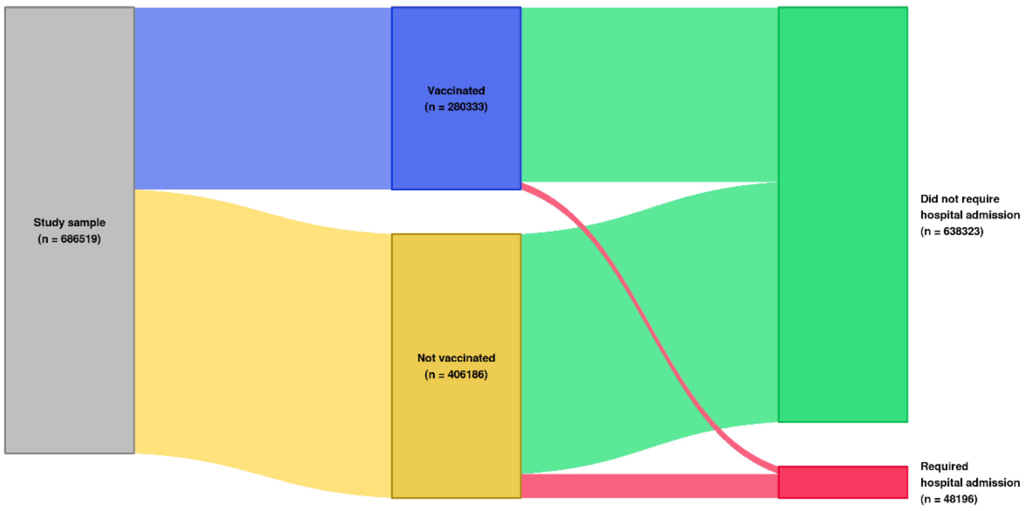

Supplementary Fig. 2 Sample distribution flows towards hospital admission, categorized by vaccination status, at the time when laboratory-confirmed cases of SARS-CoV-2 infection occur

Sample distribution flows towards ICU admission

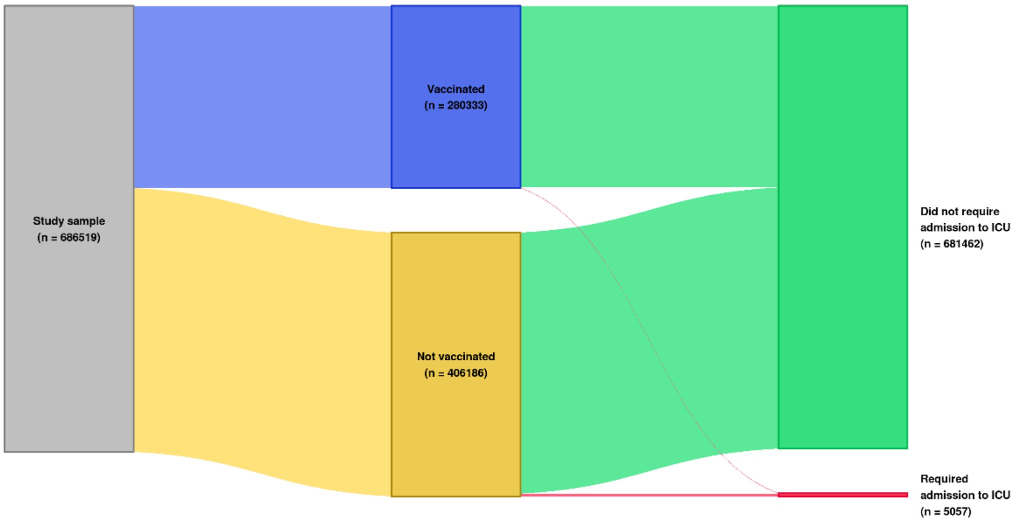

Supplementary Fig. 3 Sample distribution flows towards ICU admission, categorized by vaccination status, at the time when laboratory-confirmed cases of SARS-CoV-2 infection occur

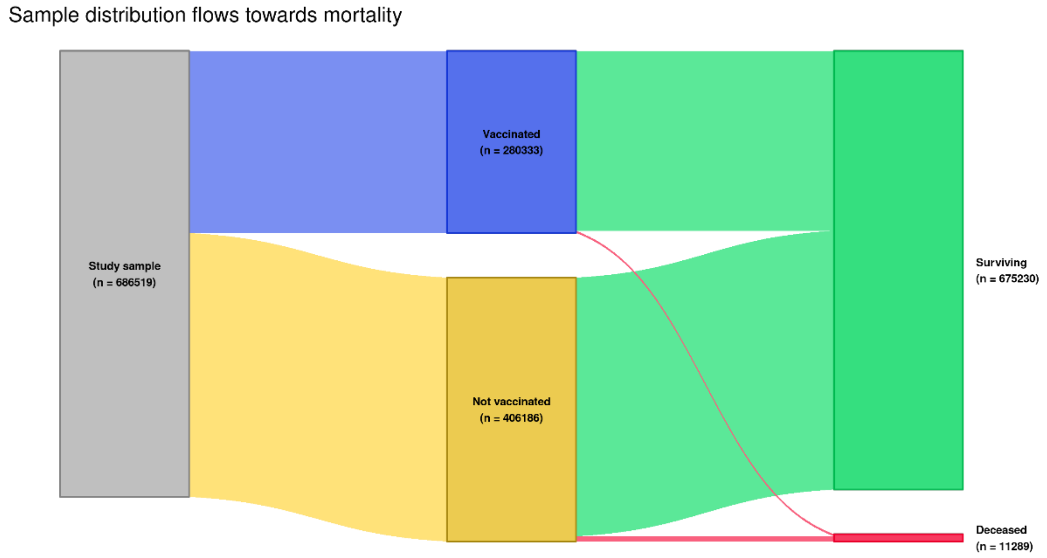

Supplementary Fig. 4 Sample distribution flows towards 30-day mortality, categorized by vaccination status, at the time when laboratory-confirmed cases of SARS-CoV-2 infection occur

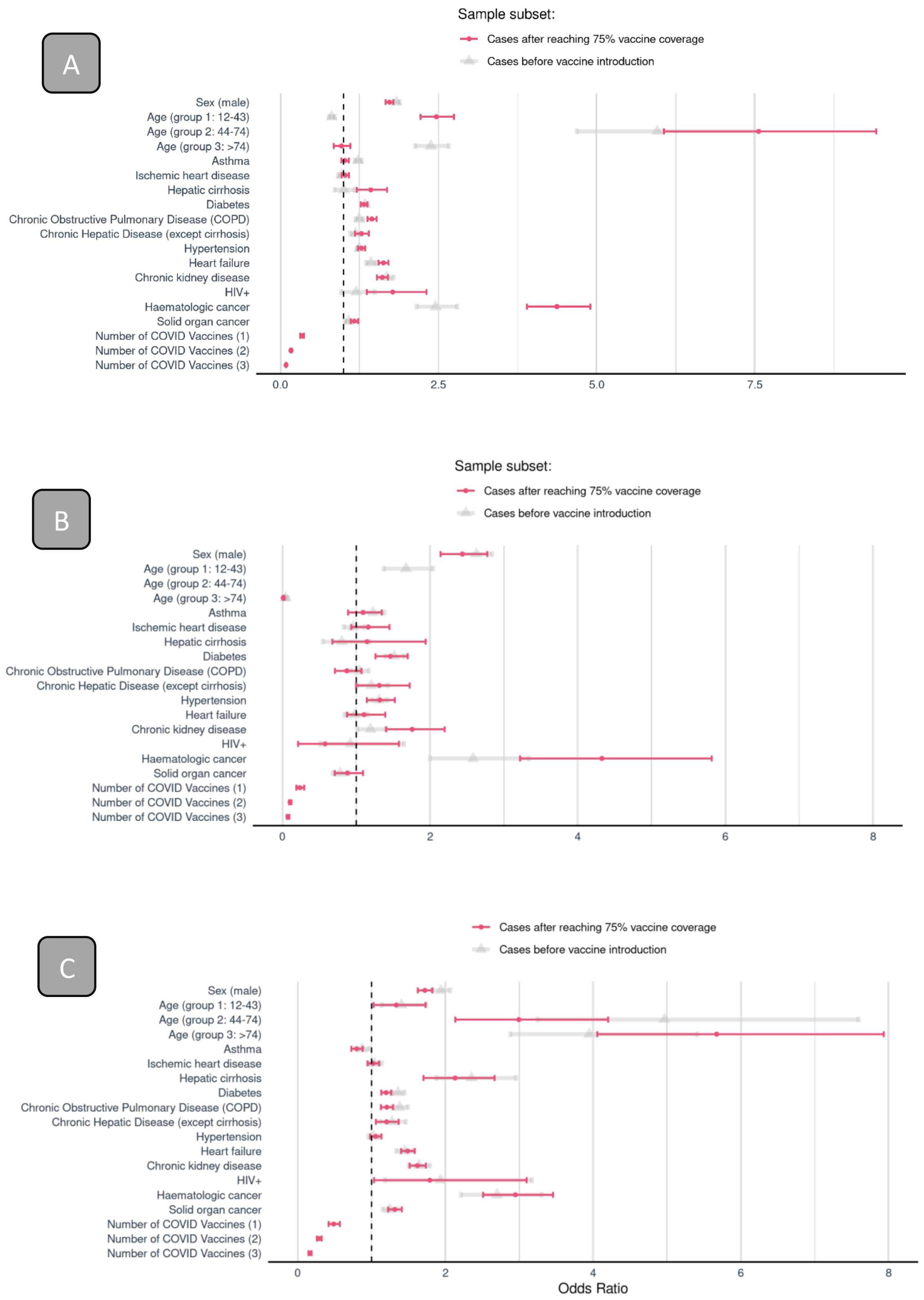

Supplementary Fig. 5 Odds ratios and confidence intervals for A. Hospital admission B. ICU admission C. 30-day mortality associated factors due to COVID-19 infection, before vaccine introduction and after reaching 75% vaccine coverage

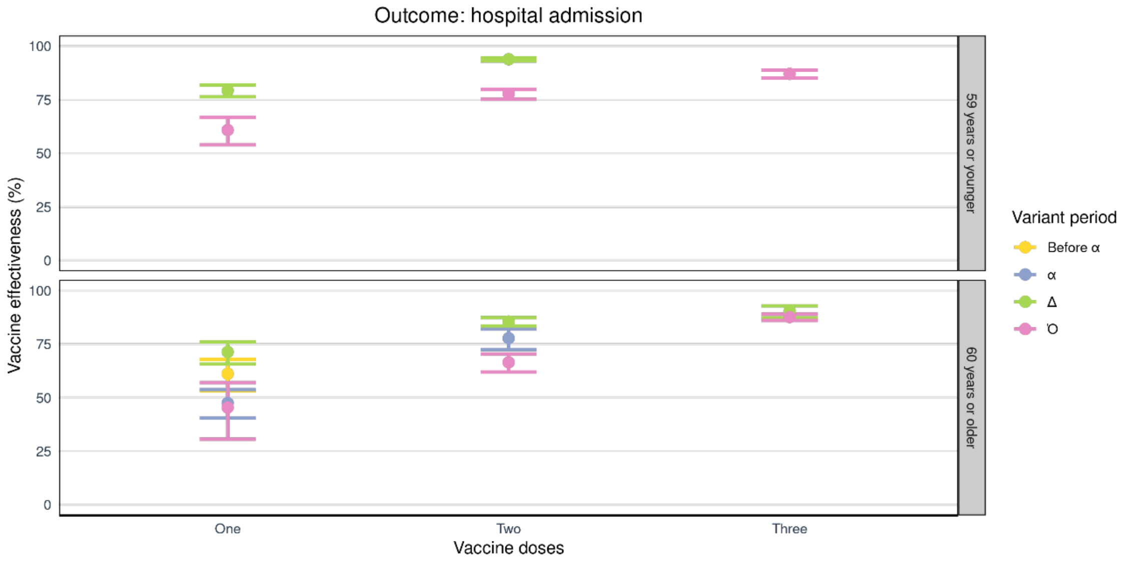

Supplementary Fig. 6 Vaccine effectiveness for hospital admission among patients aged 59 years or younger and those aged 60 years or older, stratified by vaccine doses and variant period

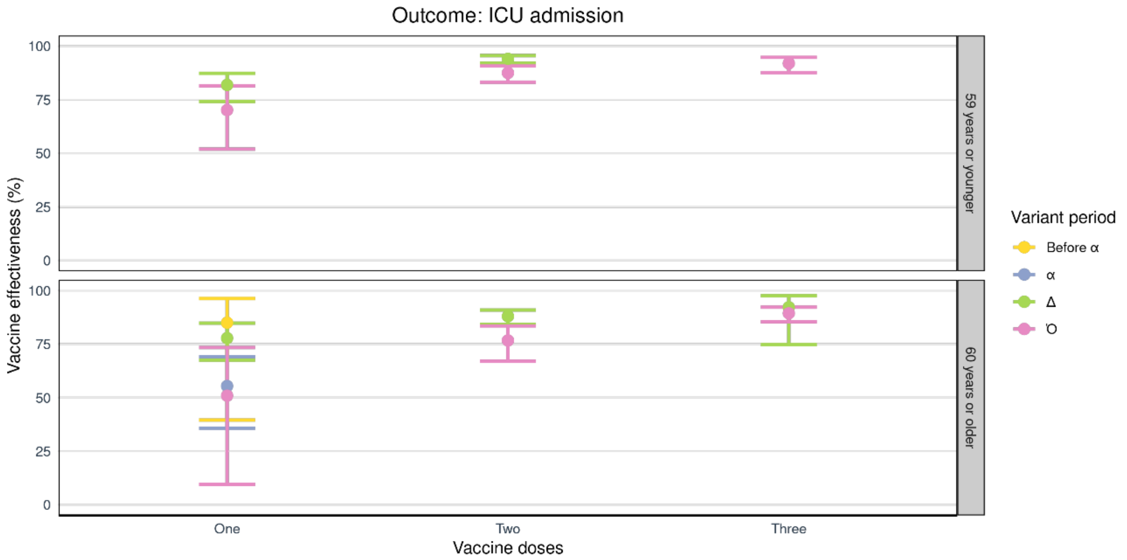

Supplementary Fig. 7 Vaccine effectiveness for ICU admission among patients aged 59 years or younger and those aged 60 years or older, stratified by vaccine doses and variant period

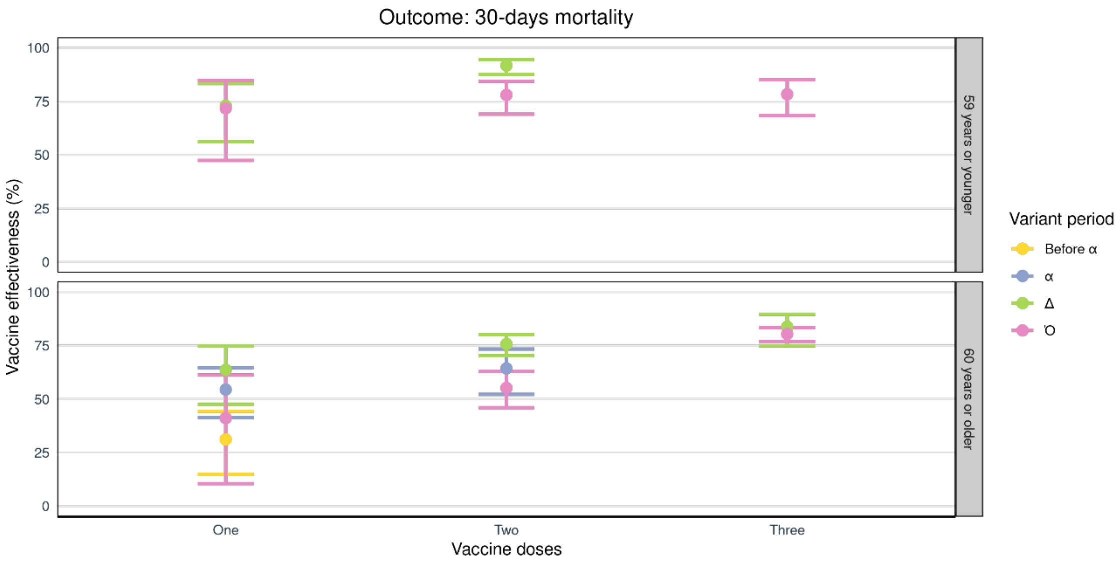

Supplementary Fig. 8 Vaccine effectiveness for 30-day mortality among patients aged 59 years or younger and those aged 60 years or older, stratified by vaccine doses and variant period

Supplementary Table 1. Diagnoses codes associated with SARS-CoV2 infection are included in the Andalusia Minimum Basic Data Set

**Diagnoses codes**	**ICD-10**
COVID-19	U07.1
Pneumonia due to SARS-associated coronavirus	J12.81
Pneumonia due to coronavirus disease 2019	J12.82
SARS-associated coronavirus as the cause of diseases classified elsewhere	B97.21
Coronavirus infection, unspecified	B34.2
Pneumonia, unspecified	J18.9
Other viral pneumonia	J12.89
Unspecified acute lower respiratory infection	J22
Acute respiratory failure, unspecified whether with hypoxia or hypercapnia	J96.00
Acute respiratory failure with hypoxia	J96.01
Acute respiratory failure with hypercapnia	J96.02
Acute and chronic respiratory failure, unspecified whether with hypoxia or hypercapnia	J96.20
Bronchiectasis with acute lower respiratory infection	J47.0
Acute and chronic respiratory failure with hypoxia	J96.21
Acute bronchospasm	J98.01
Interstitial pulmonary disease, unspecified	J84.9
Respiratory failure, unspecified, unspecified whether with hypoxia or hypercapnia	J96.90
Respiratory failure, unspecified with hypoxia	J96.91
Respiratory failure, unspecified with hypercapnia	J96.92
Other specified interstitial pulmonary diseases	J84.89
Acute upper respiratory infection, unspecified	J06.9
Respiratory disorder, unspecified	J98.9
Viral pneumonia, unspecified	J12.9

**Diagnoses codes**	**ICD-10**
Idiopathic interstitial pneumonia, not otherwise specified	J84.111
Acute bronchitis due to other specified organisms	J20.8
Idiopathic non-specific interstitial pneumonitis	J84.113
Acute interstitial pneumonitis	J84.114
Other specified diseases of upper respiratory tract	J39.8
Respiratory bronchiolitis interstitial lung disease	J84.115
